# Avian β-defensin 1 as an antibiotic-sparing supplement for preserving rooster semen quality and fertility during chilled storage

**DOI:** 10.1016/j.psj.2026.107668

**Published:** 2026-08-19

**Authors:** Ruthaiporn Ratchamak, Supakorn Authaida, Wuttigrai Boonkum, Vibuntita Chankitisakul

**Affiliations:** a The Research and Development Network Center of Animal Breeding and Omics, Khon Kaen University, Khon Kaen, Thailand; b Major of Animal Science, Department of Agricultural Technology, Faculty of Technology, Mahasarakham University, Maha Sarakham 44150, Thailand; c Department of Animal Science, Faculty of Agriculture, Khon Kaen University, Khon Kaen, Thailand

**Keywords:** Bacterial contamination, Antimicrobial peptide, Oxidative stress, Semen preservation, Artificial insemination

## Abstract

Maintaining semen quality during chilled storage is challenging due to bacterial proliferation and oxidative damage, which impair sperm function and fertility. In this study, we evaluated the effects of supplementing a conventional antibiotic and a host-derived antimicrobial peptide in a semen extender on semen quality, bacterial load, and fertility in Thai native roosters during chilled storage. Semen was diluted in an IGGKPh extender supplemented with kanamycin (31.2 µg/mL) or avian β-defensin 1 (AvBD1; 7.5 µg/mL) and stored at 5°C for 0, 24, and 48 h. Data were analyzed using repeated-measures models. At 0 h, both antimicrobial treatments increased total motility and reduced malondialdehyde (MDA) concentrations compared with the control (P < 0.05), whereas viability and acrosome integrity were unaffected. At 24 h, both treatments maintained higher total and progressive motility and acrosome integrity than the control (P < 0.05); viability was higher with AvBD1, whereas MDA concentration was lower with kanamycin. At 48 h, both antimicrobial treatments maintained higher motility, viability, and acrosome integrity and lower MDA concentrations than the control (P < 0.05). Both treatments reduced the viable bacterial load at 0 and 24 h, whereas AvBD1 produced the lowest bacterial load at 48 h (P < 0.05). Fertility was higher in both antimicrobial groups at 0 and 24 h but did not differ among treatments at 48 h. Hatchability was unaffected by antimicrobial treatment. Overall, kanamycin and AvBD1 partially mitigated, but did not prevent, storage-related sperm deterioration. AvBD1 produced broadly similar sperm-quality and fertility responses to kanamycin under controlled storage conditions, supporting its potential as an antibiotic-sparing candidate for short-term poultry semen preservation.

## Introduction

Artificial insemination (AI) is an important reproductive technology for accelerating genetic improvement and supporting the conservation of indigenous poultry genetic resources. However, the success of AI programs is frequently limited by the rapid decline in semen quality during dilution and short-term storage (Chankitisakul et al., 2022; Tvrdá et al., 2023b; Ratchamak et al., 2025). In practical production systems, rooster semen is commonly stored at chilled temperatures prior to insemination; however, sperm viability rapidly declines, limiting the effective duration.

Bacterial contamination is one of the main factors contributing to semen deterioration in poultry. Owing to the anatomical structure of the avian cloaca, semen is inevitably exposed to microorganisms during collection, which can proliferate during storage and compromise sperm function (Boone and Hughes, 1970; Mezhoud et al., 2015; Mohan et al., 2023). The presence of bacteria has been associated with reduced motility, decreased viability, and impaired fertilization capacity, thereby compromising reproductive performance (Haines et al., 2013; Ďuračka et al., 2023). Consequently, antimicrobial supplementation in semen extenders has become a routine strategy to control microbial growth and improve semen preservation. In addition to its direct effects on sperm function, bacterial contamination may also contribute to oxidative stress within semen. Bacterial metabolism and associated inflammatory responses can generate reactive oxygen species (ROS), which in turn induce lipid peroxidation of the sperm plasma membrane (Authaida et al., 2026), thereby contributing to the deterioration of sperm quality during storage.

Conventional antibiotics, such as kanamycin, are widely used for this purpose due to their effectiveness in suppressing bacterial proliferation (Sexton et al., 1980; Duracka et al., 2019; Tvrdá et al., 2023a). However, increasing concerns regarding antimicrobial resistance and regulatory restrictions on antibiotic use have prompted the search for alternative strategies (Morrell and Wallgren, 2014; Lima et al., 2024). Therefore, antimicrobial peptides (AMPs) have emerged as promising antibiotic-sparing candidates because of their direct antimicrobial activity and modes of action that differ from those of conventional antibiotics (Zhang et al., 2021; Aguilera-Puga et al., 2024). Among avian AMPs, avian β-defensin 1 (AvBD1), formerly designated gallinacin-1, was initially identified as a cysteine-rich antimicrobial peptide isolated from chicken leukocytes and shown to possess direct *in vitro* antimicrobial activity (Harwig et al., 1994). Like other native avian β-defensins, AvBD1 is a short, cationic peptide characterized by a conserved cysteine framework that stabilizes its structure through intramolecular disulfide bonds. Its cationic and amphipathic properties facilitate interactions with negatively charged bacterial surfaces and contribute to membrane-disruptive antimicrobial activity (van Dijk et al., 2008; Cuperus et al., 2013; Nguyen et al., 2021).

AvBD1 is expressed in chicken sperm, male reproductive organs, and the rooster testis, supporting its physiological relevance as a component of innate antimicrobial defense within the male reproductive system (Das et al., 2011; Watanabe et al., 2011; Anastasiadou et al., 2013, 2014). Moreover, synthetic AvBD1 variants have exhibited activity against both Gram-negative and Gram-positive bacteria and can disrupt bacterial membrane permeability, although their antimicrobial potency varies with amino acid sequence and target organism (Cadwell et al., 2017; Nguyen et al., 2021). Together, these direct antimicrobial properties and endogenous reproductive expression provide a strong biological rationale for evaluating AvBD1 as an antimicrobial supplement in poultry semen extenders.

However, direct comparative evidence between host-derived AMPs and conventional antibiotics in poultry semen extenders remains limited. Furthermore, the antimicrobial efficacy of AvBD1 may depend on peptide structure, bacterial species, microbial burden, and exposure conditions. Its effectiveness as a standalone antimicrobial under highly contaminated field conditions has not yet been established. Therefore, whether AvBD1 can effectively reduce viable bacterial burden while maintaining sperm quality and reproductive performance during chilled storage remains unclear.

Accordingly, we aimed to compare the effects of supplementing the IGGKPh extender with kanamycin or AvBD1 on sperm quality, viable bacterial load, and reproductive performance in Thai native chickens (Pradu Hang Dum) during storage at 5°C. We hypothesized that, under the controlled chilled-storage conditions evaluated, AvBD1 would reduce bacterial burden and maintain sperm function and fertility to an extent similar to kanamycin, thereby supporting its potential as an antibiotic-sparing candidate for short-term poultry semen preservation.

## Materials and methods

### Animals and management

All experimental procedures were approved by the Animal Ethics Committee of Khon Kaen University (IACUC-KKU-147/68).

Thirty Thai native roosters (Pradu Hang Dum; 78 weeks old) were housed individually in battery cages (60 × 45 × 45 cm) under an open-house system. Birds were provided with a commercial breeder diet (130 g/bird/day) and water ad libitum.

For fertility evaluation, thirty Thai native hens (Kai Mook Esan breed; 32 weeks old; laying rate >80%) were housed individually under similar conditions. Hens were fed a commercial layer diet (110 g/bird/day) with water available ad libitum.

### Semen collection and initial evaluation

Semen was collected once weekly from each rooster using the dorso-abdominal massage technique. Ejaculates were immediately transferred into 1.5 mL microtubes containing 0.1 mL of semen extender and transported to the laboratory at 22–25°C within 20 min while protected from direct sunlight. Upon arrival, ejaculates were gently mixed and evaluated individually. Only ejaculates meeting the predefined acceptance criteria—sperm concentration ≥3 × 10⁹ spermatozoa/mL, total motility ≥80%, and viability ≥90%—were included and pooled to reduce individual variation.

Pooled semen was diluted (1:3, v/v) with the respective extender according to treatment. The final sperm concentration was adjusted to approximately 400 × 10⁶ spermatozoa/dose. Diluted semen samples were cooled from 25°C to 5°C over 1 h and subsequently stored at 5°C until further analysis.

### Chemicals and media preparation

Kanamycin sulfate from Streptomyces kanamyceticus was purchased from Sigma-Aldrich (St. Louis, MO, USA; product no. K400). AvBD1 was custom synthesized by solid-phase peptide synthesis (SPPS) and supplied in a linear reduced form by Beijing Tsingke Biotech Co., Ltd. (Beijing, China; lot no. JT-211654). The peptide comprised 39 amino acids with the sequence GRKSDCFRKSGFCAFLKCPSLTLISGKCSRFYLCCKRIW and a molecular weight of 4510.42 Da. Batch purity was 95.71%, as determined by high-performance liquid chromatography, and peptide identity was verified by mass spectrometry. The lyophilized peptide was stored at −20°C until use. The batch-specific Certificate of Analysis and supplier confirmation are available upon request. All other chemicals were of analytical grade and were obtained from Sigma-Aldrich unless otherwise stated.

The IGGKPh extender was prepared according to Surai and Wishart (1996) and contained inositol (0.9 g), sodium glutamate (1.40 g), glucose (0.9 g), potassium citrate (0.14 g), sodium dihydrogen phosphate (0.21 g), and disodium hydrogen phosphate (0.98 g) per 100 mL of deionized water. The extender had a pH of 7.8 and an osmolality of 460 mOsm/kg.

All reagents were prepared under aseptic conditions. Solutions were weighed using an analytical balance, with a readability of 0.1 mg, and the extender was sterilized by filtration through a 0.22-µm syringe filter and stored at 4°C overnight prior to use.

### Experimental design, treatment allocation, and chilled storage

The study was conducted as a completely randomized design with repeated measurements over storage time with three extender treatments and four replicates per treatment. Three treatments were prepared using the IGGKPh extender described above: (1) control without antimicrobial supplementation, (2) kanamycin (31.2 µg/mL)–supplemented extender (Tvrdá et al., 2023a), and (3) avian β-defensin 1 (AvBD1; 7.5 µg/mL)–supplemented extender. The AvBD1 concentration of 7.5 µg/mL was selected based on prior evidence demonstrating effective antibacterial activity against poultry-associated bacterial strains, with no detectable cytotoxicity toward chicken HD11 cells (Nguyen et al., 2021).

Diluted semen samples were stored at 5°C and evaluated at 0, 24, and 48 h. At each storage time point, semen quality was assessed by determining sperm viability, total motility, progressive motility, acrosome integrity, and lipid peroxidation, expressed as malondialdehyde (MDA) concentration. Viable bacterial load was quantified to determine the antimicrobial effectiveness of each treatment. Fertility and hatchability were evaluated following artificial insemination using semen stored at the respective time points.

### Semen quality evaluation

Semen volume was measured using a 1 mL syringe (Nipro Co., Ltd., Phra Nakhon Si Ayutthaya, Thailand). Sperm concentration was determined using a hemocytometer after dilution with 0.9% sodium chloride and reported as ×10⁹ spermatozoa/mL.

Sperm motility was assessed under a light microscope at 400× magnification. For each sample, at least 200 spermatozoa were evaluated across a minimum of five fields. Total motility was defined as the percentage of spermatozoa exhibiting any form of movement, whereas progressive motility was defined as the percentage of spermatozoa moving actively in a forward direction.

Sperm viability was determined using eosin–nigrosin staining, as described by Authaida et al. (2024). Briefly, semen was mixed with stain, smeared on a glass slide, and air-dried. At least 300 spermatozoa were examined under a light microscope at 1,000× magnification, and viability was expressed as the percentage of live (unstained) sperm cells.

Acrosome integrity was evaluated using Coomassie blue staining, as described by Saiyamanon et al. (2025). Briefly, semen samples were fixed in 4% (w/v) paraformaldehyde, centrifuged at 1,000 × g for 5 min, and washed three times with 0.1 M ammonium acetate. The resulting pellet was used to prepare thin smears, which were stained with Coomassie blue, rinsed with deionized water, and air-dried. Slides were examined under a light microscope at 1,000× magnification, and at least 100 spermatozoa per sample were evaluated. Acrosome integrity was expressed as the percentage of spermatozoa with intact acrosomes.

### Lipid peroxidation

Lipid peroxidation was assessed by measuring MDA levels using the thiobarbituric acid (TBA) assay, as described by Ratchamak et al. (2023). Briefly, semen samples were incubated with ferrous sulfate (0.2 mM) and ascorbic acid (1 mM) at 37°C for 60 min. Subsequently, trichloroacetic acid (15% [w/v]) and thiobarbituric acid (0.375% [w/v]) were added, and the mixture was heated in a boiling water bath for 10 min. The reaction was terminated by cooling at 4°C, followed by centrifugation at 800 × g for 10 min. The absorbance of the supernatant was measured at 532 nm using a UV–Vis spectrophotometer (Specord 250 Plus, Analytik Jena, Germany). MDA concentration was expressed as µmol/mL.

### PMA-dPCR estimation of viable bacterial load

The bacterial load in semen samples was estimated using propidium monoazide (PMA) pretreatment followed by genomic DNA extraction and digital PCR as described by Nocker et al. (2009). PMA preferentially suppresses amplification of DNA from membrane-compromised bacterial cells, thereby allowing estimation of the membrane-intact bacterial or viable bacterial population.

#### PMA pretreatment

Semen samples were treated with PMA (final concentration 50 µM) to suppress amplification of DNA from non-viable bacteria. Samples were incubated in the dark for 5 min, then photoactivated under halogen light for 2 min. To prevent heat-induced damage, samples were kept on ice during light exposure. After treatment, samples were centrifuged to pellet cells prior to DNA extraction.

#### DNA extraction

Genomic DNA was extracted from semen samples using a standard lysis and precipitation protocol. Briefly, samples were lysed using SDS, sodium acetate, proteinase K, and guanidine HCl, and then incubated at 60°C. DNA was precipitated using isopropanol, washed with 75% ethanol, air-dried, and resuspended in TE buffer. DNA concentration and purity were assessed using a Qubit dsDNA High Sensitivity Assay Kit (Qubit™ 4 Fluorometer, Thermo Fisher Scientific, Singapore). Extracted DNA was stored at −20°C until analysis.

#### Digital PCR

Quantification of bacterial DNA was performed using a QIAcuity® Digital PCR system (QIAGEN, Germany) with a microbial assay targeting conserved bacterial gene sequences. Reactions were prepared according to the manufacturer’s instructions and run on a 26 K QIAcuity Nanoplate. Thermal cycling included initial activation at 95°C for 2 min, followed by 40 cycles of denaturation and annealing/extension.

Absolute bacterial counts were calculated using Poisson statistics via the QIAcuity Software Suite without the need for a standard curve. A no-template control was included in each run to monitor contamination. Results were expressed as copies/mL of semen sample.

### Artificial insemination and fertility trials

Fertility was evaluated by artificial insemination of hens once weekly using 0.4 mL of semen per hen, providing approximately 400 × 10⁶ spermatozoa per hen. Each hen was inseminated once per week for four consecutive weeks. Inseminations were performed between 15:00 and 16:00 h. Eggs were collected from Day 2 after the first insemination until Day 8 after the final insemination. Eggs were incubated under standard conditions, and fertility was determined by candling on Day 7 of incubation. Hatchability was assessed on Day 21. Fertility was expressed as the percentage of fertile eggs relative to the total number of eggs set, whereas hatchability was calculated as the percentage of hatched eggs relative to the number of fertile eggs.

### Statistical analysis

Before performing the statistical analysis for each experiment, the data were tested for normality using the Shapiro−Wilk test. The homogeneity of the residual variances was tested using Levene’s test and the data were screened for outliers. Data on semen quality, lipid peroxidation, viable bacterial load, fertility, and hatchability were analyzed using repeated measures models. For semen-quality parameters, MDA concentration, and viable bacterial load, independent semen-collection and pooling replicates were included as random effects, and storage time was treated as a repeated effect within each treatment. For fertility and hatchability, hens were randomly assigned treatment groups. An individual hen was considered the experimental unit and was included as a random subject nested within treatment. Treatment was specified as the between-subject factor, whereas storage time was specified as the within-subject repeated factor. The effects of treatment, time to storage, and their interaction mean values for each parameter were compared using Tukey’s HSD post hoc test. They were considered statistically significant at *P*< 0.05, and the results are expressed as mean values ± SEM.

## Results

The fresh semen used in this study exhibited high quality, with an average volume of 0.46 mL and a sperm concentration of 5.05 × 10⁹ spermatozoa/mL. Total and progressive motility were 96.41% and 87.47%, respectively, whereas sperm viability reached 96.55%. The initial bacterial load was relatively low (12.83 × 10⁴ copies/mL).

### *Effects of antimicrobial supplementation on semen quality during storage*

Repeated-measures analysis showed significant effects of treatment and storage time on all semen-quality parameters and MDA concentration (P < 0.05). Treatment × storage time interactions were significant for sperm viability, acrosome integrity, and MDA concentration, but not for total or progressive motility (Table 1).

**Table 1:** Effects of kanamycin and avian β-defensin 1 supplementation on rooster semen quality and viable bacterial load during storage at 5°C for 0, 24, and 48 h.

Time to storage (h)	Treatment			SEM	*P*-value		
	Control	Kanamycin	AvBD1		Treatment	Time to storage	Interaction
**Total motility (%)**							
0	92.16^b,x^	96.05^a,x^	96.24^a,x^	0.33	0.0050	<0.001	0.2584
24	80.87^b,y^	87.49^a,y^	87.67^a,y^	0.72			
48	61.00^b,z^	64.89^a,z^	66.50^a,z^	1.76			
**Progressive motility (%)**							
0	86.02^b,x^	89.43^a,x^	88.97^ab,x^	0.59	0.0045	<0.001	0.6216
24	73.01^b,y^	75.70^a,y^	75.97^a,y^	2.82			
48	37.01^b,z^	41.49^a,z^	42.82^a,z^	0.59			
**Viability (%)**							
0	95.83^x^	97.07^x^	97.72^x^	0.43	0.0172	<0.001	0.0146
24	86.07^b,y^	87.84^ab,y^	89.49^a,y^	0.41			
48	66.65^b,z^	70.14^a,z^	70.64^a,z^	0.12			
**Acrosome integrity (%)**							
0	97.82^x^	98.55^x^	98.39^x^	0.05	0.0011	<0.001	0.0104
24	92.54^b,y^	95.71^a,y^	95.76^a,y^	0.11			
48	89.03^b,z^	92.82^a,z^	93.59^a,z^	0.45			
**MDA (μmol/mL)**							
0	0.79^b,z^	0.66^a,z^	0.64^a,z^	0.01	0.0116	<0.001	0.0262
24	2.19^b,y^	1.87^a,y^	1.90^ab,y^	0.01			
48	2.82^b,x^	2.74^a,x^	2.73^a,x^	0.01			
**Total bacterial load (×10⁴ copies/mL)**							
0	14.96^b,y^	11.90^a,y^	11.56^a,y^	3.97	0.0002	<0.001	0.0788
24	16.38^b,x^	10.93^a,x^	10.65^a,x^	2.78			
48	18.23^c,x^	16.28^b,x^	15.39^a,x^	1.83			

Values are presented as least-squares means with SEM. Different superscript letters (a–c) within the same row indicate significant differences among treatments at the same storage time, whereas different superscript letters (x–z) within the same treatment column for each variable indicate significant differences among storage times (P < 0.05). P-values represent the main effects of treatment and storage time and their interaction from the repeated-measures analysis. SEM = standard error of the mean; AvBD1 = avian β-defensin 1; MDA = malondialdehyde.

At 0 h, total motility was higher in the kanamycin and AvBD1 groups than in the control (P < 0.05). Progressive motility was higher with kanamycin than in the control (P < 0.05), whereas the AvBD1 group did not differ from the control or kanamycin groups. Viability and acrosome integrity were similar among treatments. MDA concentrations were lower in both antimicrobial-supplemented groups than in the control (P < 0.05).

At 24 h, total and progressive motility and acrosome integrity were higher in both antimicrobial-supplemented groups than in the control (P < 0.05). Viability was higher with AvBD1 than in the control (P < 0.05), whereas the kanamycin group did not differ from the control or AvBD1 groups. MDA concentration was lower in the kanamycin group than in the control group (P < 0.05), whereas the AvBD1 group did not differ from the control or kanamycin groups.

At 48 h, both antimicrobial treatments resulted in higher total and progressive motility, viability, and acrosome integrity and lower MDA concentrations than the control (P < 0.05). These parameters did not differ between kanamycin and AvBD1.

### *Effects of antimicrobial supplementation on viable bacterial load*

Repeated-measures analysis showed significant effects of treatment and storage time on the viable bacterial load (P < 0.05), whereas the treatment × storage time interaction was not significant (Table 1). At 0 and 24 h, the bacterial load was higher in the control than in the kanamycin and AvBD1 groups (P < 0.05), with no difference between the antimicrobial treatments. At 48 h, all treatments differed (P < 0.05), with the highest bacterial load in the control, followed by kanamycin and AvBD1.

### *Effects of antimicrobial supplementation on fertility and hatchability*

Repeated-measures analysis showed significant effects of treatment and storage time on fertility (P < 0.05), whereas the treatment × storage time interaction was not significant (Table 2). At 0 and 24 h, fertility was higher in the kanamycin and AvBD1 groups than in the control (P < 0.05), with no difference between the antimicrobial treatments. At 48 h, fertility did not differ among treatment groups.

**Table 2:** Effects of kanamycin and avian β-defensin 1 supplementation on fertility and hatchability of fertile eggs following artificial insemination with semen stored at 5°C for 0, 24, and 48 h.

Time to storage (h)	Treatment			SEM	*P*-value		
	Control	Kanamycin	AvBD1		Treatment	Time to storage	Interaction
**Total number of eggs (n)**							
	442	448	437				
**Fertility (%)**							
0	73.61^b,x^	78.38^a,x^	79.70^a,x^	0.67	0.0101	<0.001	0.6461
24	65.41^b,y^	73.77^a,y^	74.95^a,y^	1.95			
48	49.18^z^	53.62^z^	54.86^z^	6.24			
**Hatchability of fertile eggs (%)**							
0	90.60^y^	91.38^y^	92.72^y^	1.96	0.3917	0.0019	0.1600
24	92.67^xy^	97.26^xy^	93.40^xy^	1.00			
48	97.51^x^	96.36^x^	95.57^x^	1.62			

Total egg numbers are presented as counts, whereas fertility and hatchability are presented as least-squares means with SEM. Different superscript letters (a, b) within the same row indicate significant differences among treatments at the same storage time, whereas different superscript letters (x–z) within the same column indicate significant differences among storage times within the same treatment (P < 0.05). P-values represent the main effects of treatment and storage time and their interaction from the repeated-measures analysis. SEM = standard error of the mean; AvBD1 = avian β-defensin 1.

Hatchability was affected by storage time (P < 0.05), but not by treatment or the treatment × storage time interaction. Hatchability was higher at 48 h than at 0 h, whereas values at 24 h were intermediate.

## Discussion

Bacterial contamination is a major driver of semen deterioration during chilled storage, directly impairing sperm motility, membrane integrity, and fertilizing capacity (Mohan et al., 2023; Tvrdá et al., 2023b; Pimpa et al., 2024). In the present study, both kanamycin and AvBD1 reduced viable bacterial load and partially mitigated storage-related declines in sperm motility, viability, and acrosome integrity compared with the unsupplemented control. However, sperm quality declined progressively with storage time in all treatments, and the effects on viability, acrosome integrity, and MDA concentration varied with storage duration. Lower MDA concentrations in antimicrobial-supplemented semen at selected storage times suggest an association between microbial control and reduced lipid peroxidation, although a direct antioxidant effect cannot be inferred. Fertility was higher in both antimicrobial-supplemented groups at 0 and 24 h but not at 48 h. Collectively, these findings indicate that microbial control contributed to, but did not fully prevent, time-dependent deterioration of chilled rooster semen.

### *Distinct antimicrobial mechanisms of kanamycin and AvBD1*

Although kanamycin and AvBD1 produced broadly similar sperm-preserving effects, their antimicrobial mechanisms differ fundamentally. Kanamycin inhibits bacterial protein synthesis through interaction with the bacterial ribosome, thereby suppressing microbial growth in semen extenders (Tvrdá et al., 2023a,b). In contrast, the AvBD1 evaluated in the present study was a 39-amino-acid, cationic, cysteine-rich peptide supplied in a linear reduced form. Its membrane-active properties appear to depend on the combined distribution of cationic and hydrophobic residues (Harwig et al., 1994; Cadwell et al., 2017). The enrichment of lysine and arginine residues among hydrophobic residues results in a reported net charge of +8, an isoelectric point of 10.0, and moderate hydrophobicity (Nguyen et al., 2021). These properties may promote electrostatic binding to negatively charged bacterial surfaces, followed by membrane insertion and permeabilization. These sequence-based properties may also provide a rational basis for designing shorter or modified AvBD1 analogs with improved antimicrobial selectivity, stability, and sperm compatibility.

Previous studies support this membrane-active mechanism. Synthetic AvBD1 variants exhibited activity against both Gram-negative and Gram-positive bacteria and increased bacterial membrane permeability, although their potency varied with peptide sequence and bacterial target (Cadwell et al., 2017). Nguyen et al. (2021) likewise demonstrated direct antimicrobial activity against several poultry-associated bacterial species. These findings indicate that AvBD1 can act directly on bacterial cells in addition to its physiological role in innate defense.

The reduction in viable bacterial load in AvBD1-treated semen is consistent with the previously reported antimicrobial properties of AvBD1. However, the present study did not assess bacterial membrane damage or characterize treatment-related changes in bacterial community composition. Therefore, the current results cannot identify the bacterial taxa most susceptible to AvBD1 or directly confirm its membrane-active mechanism in rooster semen. In a previous study, our group identified 14 bacterial taxa in untreated fresh semen from Thai native roosters, including 11 Gram-negative and 3 Gram-positive taxa, and found that higher bacterial load was associated with increased MDA concentrations and reduced sperm motility and viability (Authaida et al., 2026). However, because those data were obtained from different birds and untreated samples, they cannot determine which taxa were affected by AvBD1 or kanamycin in the present experiment. The current findings should therefore be interpreted as treatment-related differences in overall viable bacterial load rather than taxon-specific antimicrobial activity. Future studies integrating PMA-dPCR with culture-based identification or 16S rRNA sequencing are needed to characterize the bacterial populations affected during chilled storage.

### *Physiological relevance and sperm compatibility of AvBD1*

The potential value of AvBD1 in semen preservation is supported not only by its direct antimicrobial properties but also by its endogenous association with the avian male reproductive system. AvBD1 has been detected in chicken sperm and male reproductive tissues, including the epididymis and testis, supporting its physiological relevance as a component of innate antimicrobial defense in the reproductive system (Das et al., 2011; Watanabe et al., 2011; Anastasiadou et al., 2014). However, endogenous occurrence alone does not establish the safety or effectiveness of exogenous AvBD1 supplementation in semen extenders.

The concentration of 7.5 µg/mL was selected based on Nguyen et al. (2021), who demonstrated antibacterial activity against several poultry-associated bacterial strains without detectable cytotoxicity toward chicken HD11 cells over the tested concentration range. In the present study, AvBD1 did not reduce sperm viability or acrosome integrity compared with kanamycin at any storage time and maintained both parameters above the unsupplemented control at 24 and 48 h. These findings provide no evidence of overt sperm toxicity at the concentration evaluated.

Nevertheless, only one AvBD1 concentration was examined, and the cytotoxicity data reported by Nguyen et al. (2021) were obtained using chicken macrophage cells rather than spermatozoa. Mitochondrial membrane potential, ATP production, membrane fluidity, and other sensitive indicators of subcellular toxicity were also not evaluated. Therefore, the present findings support apparent sperm compatibility at 7.5 µg/mL but do not establish an optimal concentration or a complete safety profile for sperm. Further dose–response studies combining bacterial inhibition with detailed sperm functional assessments are required to define the effective and safe concentration range of AvBD1 in poultry semen extenders.

The structural and functional stability of AvBD1 in the IGGKPh extender was not directly evaluated. Although antimicrobial peptides may be susceptible to proteolytic degradation, the marked decline in progressive motility between 24 and 48 h occurred across all treatments rather than specifically in the AvBD1 group. At 48 h, AvBD1-treated semen still exhibited higher progressive motility, viability, and acrosome integrity and lower MDA concentrations than the unsupplemented control, while showing the lowest viable bacterial load. This pattern does not support attributing the decline in progressive motility specifically to AvBD1 exhaustion or degradation. Nevertheless, the bacterial load response does not confirm that AvBD1 remained structurally intact or establish its half-life, because the suppression of bacteria may partly reflect activity exerted earlier during storage. Partial degradation or a gradual loss of functional activity therefore cannot be excluded. Future studies should quantify intact AvBD1 and its degradation products over time using LC-MS or HPLC, and assess residual antimicrobial activity alongside sperm motility and mitochondrial function.

### *Role of oxidative stress in semen deterioration during storage*

Lipid peroxidation is a major contributor to sperm damage during storage, particularly in avian species whose sperm membranes are rich in polyunsaturated fatty acids (Partyka et al., 2012, Partyka et al., 2023; Mussa et al., 2021). Oxidative damage compromises membrane integrity and cellular function, ultimately reducing sperm motility and viability.

Oxidative stress in semen may also be associated with microbial contamination. Gram-negative bacteria can promote ROS generation through lipopolysaccharide-mediated inflammatory and oxidative pathways, while bacterial metabolism and toxic by-products may further disrupt the oxidative balance of the seminal environment (Reddy et al., 2006; He et al., 2017; Tvrdá et al., 2022). Consistent with this relationship, our previous study showed that bacterial load was positively associated with MDA concentration and negatively associated with sperm motility and viability in rooster semen (Authaida et al., 2026).

In the present study, the significant treatment × storage time interaction for MDA indicated that the effect of antimicrobial supplementation on lipid peroxidation varied during storage. MDA concentrations were lower in both antimicrobial-supplemented groups than in the control at 0 and 48 h, whereas at 24 h, kanamycin resulted in a lower MDA concentration than the control and AvBD1 showed an intermediate response. These findings support an association between microbial control and reduced lipid peroxidation but do not demonstrate a direct antioxidant effect of AvBD1.

At 48 h, AvBD1 resulted in a lower viable bacterial load than kanamycin, but this additional reduction was not accompanied by further improvements in motility, viability, acrosome integrity, or MDA concentration. This apparent disconnect suggests that the relationship between bacterial load and sperm function is not strictly linear. Both antimicrobial treatments may have reduced the microbial challenge below a biologically damaging threshold, beyond which further bacterial suppression provided limited additional protection. Moreover, membrane aging, metabolic depletion, and storage-associated mitochondrial and oxidative dysfunction may have become increasingly important during prolonged storage. Although the absence of poorer sperm-quality outcomes with AvBD1 provides no evidence of overt cytotoxicity, subtle mitochondrial effects cannot be excluded because mitochondrial membrane potential, ATP production, and mitochondrial oxidative stress were not directly assessed.

### *Functional implications for fertility and hatchability*

Both kanamycin and AvBD1 resulted in higher fertility than the unsupplemented control at 0 and 24 h, supporting an association among microbial control, maintenance of sperm function, and fertilization success. However, fertility did not differ among treatments at 48 h, despite the higher sperm-quality parameters and lower bacterial loads observed in the antimicrobial-supplemented groups.

By 48 h, progressive motility had declined to approximately 37–43% across treatments, potentially limiting sperm transport and retention within the sperm-storage tubules and consequently reducing fertilizing capacity (Kheawkanha et al., 2021). Fertility values at this time remained within the range previously reported for chilled rooster semen stored for a similar duration (Bui et al., 2021; Authaida et al., 2024; Kheawkanha et al., 2025). The concurrent increases in MDA concentration and viable bacterial load, compared with earlier storage times, further indicate that microbial control alone was insufficient to prevent cumulative storage-related deterioration.

Hatchability of fertile eggs varied with storage time but was not affected by antimicrobial treatment. Thus, neither kanamycin nor AvBD1 appeared to compromise embryonic development under the conditions evaluated. The relatively high hatchability observed at 48 h should be interpreted cautiously because it was calculated only from fertile eggs and therefore does not offset the decline in fertility associated with prolonged semen storage.

### *Potential and translational constraints of AvBD1 as an antibiotic-sparing supplement*

Previous studies have demonstrated direct antimicrobial activity of AvBD1 against both Gram-negative and Gram-positive bacteria (Harwig et al., 1994; Cadwell et al., 2017; Nguyen et al., 2021). Consistent with these findings, AvBD1 reduced the viable bacterial load and produced sperm-preserving and fertility outcomes broadly similar to those of kanamycin under the controlled chilled-storage conditions evaluated. At 48 h, AvBD1 resulted in a lower bacterial load than kanamycin, although this additional reduction did not provide further improvement in sperm quality or fertility. These findings support AvBD1 as a promising antibiotic-sparing candidate for short-term poultry semen preservation rather than as a superior replacement for conventional antibiotics.

However, the present findings do not establish that AvBD1 can universally replace conventional antibiotics as a standalone antimicrobial. The initial bacterial load was relatively low, and deliberately contaminated semen, highly contaminated field samples, and pathogen-specific challenges were not evaluated. In addition, the absence of an AvBD1–antibiotic combination prevents conclusions regarding potential additive or synergistic effects. The present results should therefore be regarded as proof of concept rather than validation for universal standalone use. Further studies should evaluate pathogen-specific activity, dose–response relationships, peptide stability, sperm-cell safety, and whether AvBD1 is more effective alone under low-to-moderate contamination or as part of a reduced-antibiotic preservation system.

Commercial translation of AvBD1 may be primarily limited by production costs and formulation stability. The AvBD1 evaluated here was a 39-amino-acid, 4.51-kDa peptide synthesized in a linear reduced form and therefore did not require controlled disulfide pairing. Nevertheless, high-purity synthesis, purification, quality control, shelf life, proteolytic stability, and cost per insemination dose may limit routine application. Accordingly, commercial-scale use cannot yet be supported. Shorter, defined analogs or standardized lower-molecular-weight peptide preparations may reduce production costs and improve formulation and scalability; however, no universal molecular-weight range can be recommended, as biological activity depends not only on peptide size but also on amino acid sequence, charge density, hydrophobicity, amphiphilicity, conformation, and proteolytic stability. Increased cationicity and hydrophobicity may enhance bacterial membrane disruption but may also increase nonspecific interactions with sperm plasma, acrosomal, and mitochondrial membranes. Future analog design should therefore identify the minimum sequence required for antimicrobial activity while simultaneously evaluating potential antioxidant, anti-inflammatory, or metabolic regulatory activities, structural stability, manufacturability, and sperm-specific cytotoxicity.

## Conclusions

Supplementation of the IGGKPh extender with kanamycin or AvBD1 reduced the viable bacterial load and partially mitigated storage-related declines in sperm motility, viability, acrosome integrity, and oxidative status compared with the unsupplemented control. Both antimicrobial treatments were associated with higher fertility at 0 and 24 h; however, sperm quality deteriorated substantially in all treatments by 48 h, and fertility no longer differed among groups. Under the controlled conditions evaluated, AvBD1 produced broadly similar sperm-quality and fertility responses to kanamycin, supporting its potential as an antibiotic-sparing candidate rather than an established replacement for conventional antibiotics. Further studies are required to determine its dose–response relationship, stability and susceptibility to proteolytic degradation in stored semen, production cost, and scalability before field-scale application.

## Author Contribution

Ruthaiporn Ratchamak contributed substantially to the study conception and design, methodology, data acquisition and curation, formal analysis, interpretation of the results, visualization, and manuscript preparation.Supakorn Authaida contributed substantially to the study design and methodology, data acquisition and interpretation, and manuscript preparation.Wuttigrai Boonkum contributed substantially to data analysis, data curation, interpretation of the results, and manuscript preparation.Vibuntita Chankitisakul contributed substantially to the study conception and design, validation and interpretation of the results, supervision, resources, project administration, funding acquisition, and manuscript preparation.All authors participated in drafting and/or critically reviewing the manuscript, reviewed and approved the final version for publication, and agree to be accountable for the accuracy and integrity of the work.

## Disclosures

The authors declare that they have no known competing financial interests or personal relationships that could have appeared to influence the work reported in this paper.
